# Successful Treatment of Locally Advanced Microsatellite Instability-High Ascending Colon Cancer Using an Immune Checkpoint Inhibitor without Extensive Resection: A Case Report

**DOI:** 10.70352/scrj.cr.25-0007

**Published:** 2025-04-25

**Authors:** Taiki Nabekura, Yu Sato, Nobuyuki Hiruta, Natsumi Kitahara, Yuki Moriyama, Kengo Kadoya, Ayami Sato, Kotaro Wakamatsu, Masaru Tsuchiya

**Affiliations:** 1 Department of Surgery, Toho University Sakura Medical Center, Sakura, Chiba, Japan; 2 Department of Surgical Pathology, Toho University Sakura Medical Center, Sakura, Chiba, Japan

**Keywords:** colorectal cancer, immune checkpoint inhibitors, immunotherapy, biomarkers

## Abstract

**INTRODUCTION:**

Colorectal cancer is a prevalent malignancy that necessitates personalized chemotherapy, especially with the advent of molecular-targeted drugs and immune checkpoint inhibitors. In Japan, immune checkpoint inhibitors have been approved for unresectable advanced and recurrent colorectal cancer; however, their use in preoperative therapy for colorectal cancer has not yet been approved. Globally, neoadjuvant immunotherapy has demonstrated promising outcomes in colorectal cancer cases with high immunogenicity, including microsatellite instability-high and deficient mismatch repair.

**CASE PRESENTATION:**

We report a case of a microsatellite instability-high, clinically unresectable, locally advanced ascending colon cancer treated with immune checkpoint inhibitors, which showed significant tumor shrinkage, facilitating standard surgery while avoiding adjunct organ resection. The patient, a 70-year-old male, experienced chronic abdominal pain and diarrhea. Lower gastrointestinal endoscopy and computed tomography confirmed a diagnosis of ascending colon cancer with suspected invasion into the descending duodenum. Although curative resection was technically feasible with pancreatoduodenectomy, neoadjuvant chemotherapy was selected to reduce tumor size, considering the patient’s overall condition. Companion diagnostics revealed microsatellite instability-high status and *BRAF*^*V600E*^ mutation, leading to the initiation of chemotherapy combined with an immune checkpoint inhibitor (pembrolizumab). Subsequently, prolonged pembrolizumab administration was challenging due to suspected immune-related adverse events, including diarrhea and pruritus. However, significant tumor reduction was observed during a follow-up computed tomography scan, facilitating surgery approximately 6 months after treatment initiation. The perioperative period was uneventful, and the patient was discharged on the eighth day after operation. The final pathological results revealed complete tumor disappearance (histological effect of chemotherapy: Grade 3).

**CONCLUSIONS:**

This case highlights the potential of neoadjuvant immunotherapy in reducing surgical invasiveness in patients with colorectal cancer.

## Abbreviations

ADL
: activities of daily living
cCR
: clinical complete response
CoDx
: companion diagnostics
CRC
: colorectal cancer
dMMR
: deficient mismatch repair
ICI
: immune checkpoint inhibitor
irAE
: immune-related adverse event
MSI
: microsatellite instability
W&W
: watch-and-wait

## INTRODUCTION

CRC is one of the most prevalent cancers in the Japanese population, ranking among the highest in both incidence and mortality rates.^1)^

The advancement of chemotherapy has led to a more personalized approach, with the advent of molecular targeted drugs and ICIs using biomarkers, in addition to cytotoxic anticancer agents. Key biomarkers in CRC, including MSI, *RAS*, and *BRAF*, display mutation positivity rates of 4%, 47%, and 7%, respectively, in cases of unresectable CRC within the Japanese population.^2,3)^

In Japan, immunotherapy for unresectable, advanced, and recurrent CRC has been approved by the clinical guidelines of the Japanese Society for CRC and is covered by the National Health Insurance based on the KEYNOTE-177 trial. However, the use of immunotherapy as preoperative chemotherapy for resectable CRC was not approved until April 2024.

Globally, several clinical trials have evaluated the efficacy of neoadjuvant chemotherapy for CRC with high immunogenicity, such as MSI-high and dMMR. These trials have demonstrated high response rates and validated the safety of neoadjuvant chemotherapy.^4–6)^

In this study, we report a case of locally advanced ascending colon cancer positive for the MSI-high/*BRAF*^*V600E*^ mutation, wherein tumor shrinkage was achieved upon ICI administration. The patient experienced a complete pathological response, avoiding resection of the adjacent organs. This case has been presented along with a review of relevant literature.

## CASE PRESENTATION

### Primary complaint

A 70-year-old male presented with abdominal pain and diarrhea, persisting for several months.

### History of present complaint

He was diagnosed with ascending colon cancer in August 2023, and was referred to our department for close examination and treatment.

### Past medical history

Hypertension, gout, and cholelithiasis.

### Medications

Loxoprofen and teprenone.

### Allergies

None.

### Social history

Current smoker (Brinkmann index 750, instructed to quit smoking during the first visit) and habitual drinker.

### Physical examination

Height, 172 cm; weight, 69 kg; body mass index 23.3 kg/m^2^. The Eastern Cooperative Oncology Group Performance Status score was 1. The patient ambulated with a cane, and was alert and oriented. The abdomen was flat and soft with no tenderness on palpation.

### Laboratory results

#### Blood test

C-reactive protein, 0.25 mg/dL; total protein, 7.4 g/dL; albumin, 3.7 g/dL, aspartate aminotransferase, 29 U/L; alanine aminotransferase, 28 U/L; lactate dehydrogenase, 229 U/L; alkaline phosphatase, 83 U/L; gamma-glutamyl 21 U/L; cholinesterase, 260 U/L; total bilirubin, 0.6 mg/dL; blood urea nitrogen, 14.5 mg/dL; creatinine, 1.25 mg/dL; sodium, 141 mEq/L; potassium, 4.2 mEq/L; chloride, 107 mEq/L; calcium, 9.9 mg/dL; glucose, 117 mg/dL; glycated hemoglobin, 6.1%; white blood cell count, 8240/μL; red blood cell count, 411/μL; hemoglobin, 12.7 g/dL; hematocrit, 39.9%; platelet, 34.8 × 10^5^/μL; neutrophil, 70.4%; lymphocyte, 21.2%; prothrombin time-international normalized ratio, 1.05; activated partial thromboplastin time, 31.3 s; carcinoembryonic antigen, 9.4 ng/mL; and cancer antigen 19-9, 114.6 U/mL.

#### Cardiopulmonary function

Electrocardiogram, normal sinus rhythm; vital capacity percentage, 108.3% (3.66 L); forced expiratory volume % in 1 second, 77.4%.

### Imaging examinations

#### Lower gastrointestinal endoscopy

**Fig. 1:**
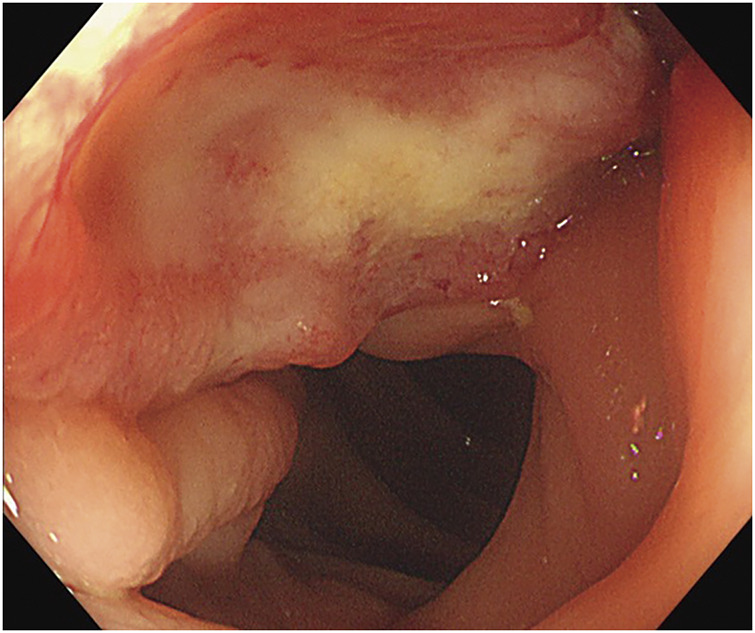
A 1/3 circumscribed, Type II tumor was detected in the ascending colon. The pathology examination result of the biopsy sample was group 5, adenocarcinoma (tub2 >por >muc).

This revealed a 1/3 circumscribed, Type II tumor in the ascending colon (**Fig. 1**). In addition, pedunculated polyps (8–10 mm) were observed in the ascending colon and sigmoid region of the rectum.

#### Upper gastrointestinal endoscopy

**Fig. 2:**
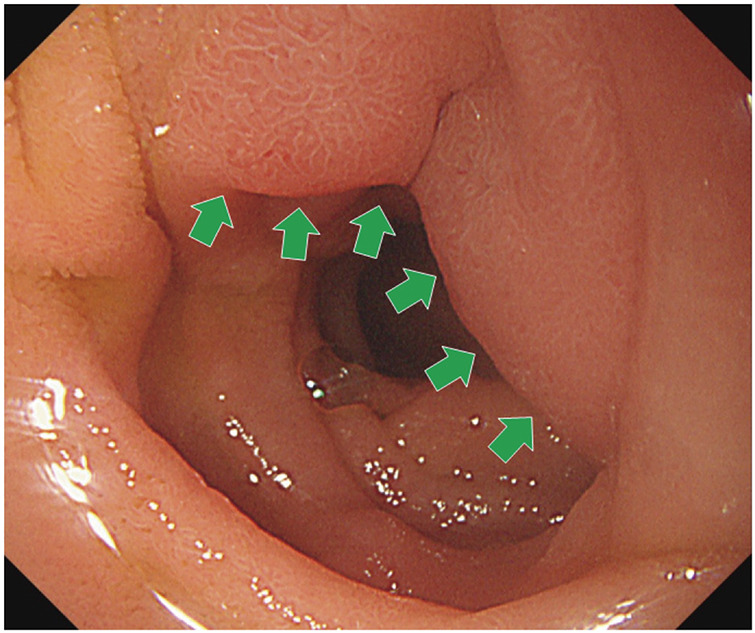
Findings of upper gastrointestinal endoscopy. Green arrows indicate the area where extramural compression was observed.

We observed atrophic gastritis (C3) and an ulcer scar (S2) in the bulb of the duodenum. There was extramural compression of the descending duodenal leg; however, no direct tumor invasion of the ascending colon was found (**Fig. 2**).

#### CT

**Fig. 3:**
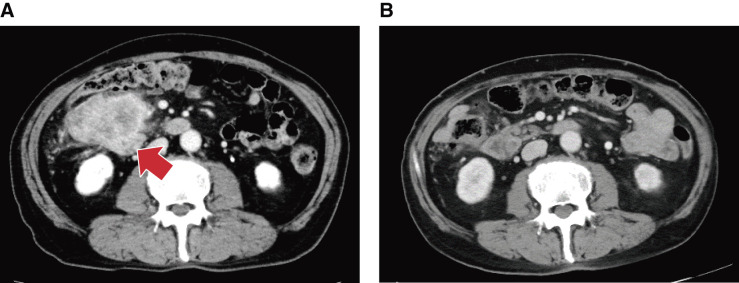
Comparison of pre- and post-immunotherapy on CT scan. (**A**) Duodenal invasion by the ascending colon cancer. The descending part of the duodenum is at the tip of the red arrow. (**B**) The tumor has shrunk significantly and a gap was formed between the tumor and the duodenum.

We observed significant wall thickening and a possible extramural invasion in the ascending colon, suggesting infiltration into the descending duodenum (**Fig. 3A**). Furthermore, enlargement of the 2 regional lymph nodes were observed. There was no evidence of distant metastasis.

#### Histological diagnosis of biopsy tissues

**Fig. 4:**
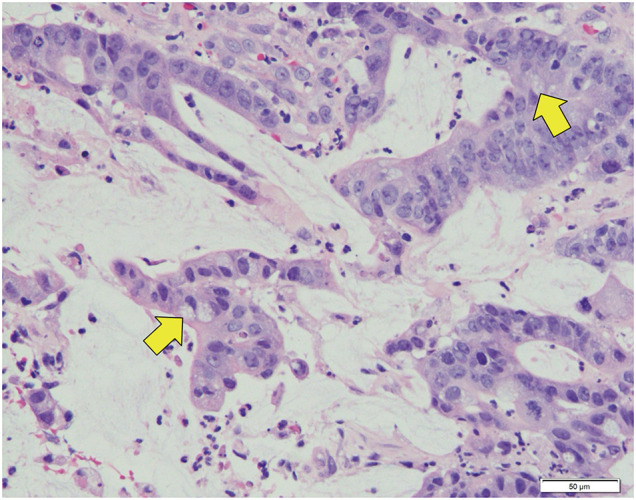
Histopathological examination of the biopsy tissue reveals moderately differentiated adenocarcinoma characterized by a fused tubular pattern of atypical, well-defined epithelium with notable mucus production (indicated by yellow arrows). The mucus fills the glandular ducts.

Colon cancer, group 5 adenocarcinoma (tub2 >por >muc). A moderately differentiated adenocarcinoma with clear epithelial-fused tubular arrangements and cribriform patterns was observed. Some parts showed progression from moderate to poorly differentiated adenocarcinoma, suggesting a transition to mucinous carcinoma (**Fig. 4**).

### Clinical diagnosis and medical treatment planning

The patient was diagnosed with cT4bN1aM0, clinical Stage IIIb CRC. Direct cancer invasion or adhesion to the descending part of the duodenum was suspected, suggesting potential resectability through combined pancreaticoduodenectomy for ascending colon cancer. However, considering the patient’s limited ADL, which were restricted to ambulation with a cane, we determined that the combined pancreaticoduodenectomy would be highly invasive. Therefore, chemotherapy was selected as the treatment for the clinically unresectable, locally advanced CRC.

### Treatment course and processes leading to conversion surgery

A definitive diagnosis of adenocarcinoma was made at the referring hospital. However, CoDx were not performed as that hospital did not specialize in gastrointestinal oncology; thus, the previous doctor sent a biopsy specimen to a specialized pathology facility. Since retrieving the biopsy specimen from the hospital after evaluation at the specialized facility was expected to take time and the adequacy of the tissue collected for CoDx was uncertain, a repeat examination was conducted at our hospital. Considering the anticipated delay in performing lower gastrointestinal endoscopy and obtaining biopsy specimens for CoDx at our institution, early initiation of treatment was prioritized. Therefore, mFOLFOX6 chemotherapy was initiated, with a preplanned strategy to adjust the therapeutic regimen based on the subsequent CoDx results. Outpatient treatment with mFOLFOX6 was initiated from Day 15 (the first visit to our hospital was considered as Day 1). CoDx conducted during the biopsy at our hospital revealed that the patient had *RAS* wild-type, *BRAF*^*V600E*^ mutation, and MSI-high status following treatment initiation. Pembrolizumab (200 mg) was administered on Days 29 and 50, according to the algorithm outlined in the Colorectal Cancer Treatment Guidelines 2022 of the Japanese Society for Cancer of the Colon and Rectum.

**Fig. 5:**
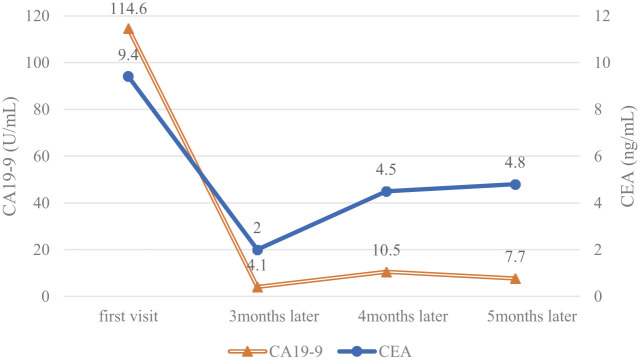
Tumor marker trends in this case.

Subsequently, the patient developed irAEs, including diarrhea (Grade 3), pruritus (Grade 2), weight loss (Grade 2), and dysgeusia (Grade 2) (all grade evaluations were based on the Common Terminology Criteria for Adverse Events v5.0), with hospitalization on Day 86. Steroid pulse therapy was initiated, and the patient was discharged on Day 94. Following discharge, oral steroids were gradually tapered in an outpatient setting. Pembrolizumab was readministered on Day 106 after the patient’s symptoms improved. However, diarrhea (Grade 2) and pruritus (Grade 2) exacerbated. A CT scan conducted on Day 141 revealed significant tumor shrinkage, with no duodenal infiltration (**Fig. 3B**), and no signs of distant metastases. The tumor marker levels, which were elevated at the initial visit, remained negative (**Fig. 5**). Due to the significant decrease in the patient’s QOL caused by irAEs, continued pembrolizumab administration was deemed unfeasible. Based on these findings and following discussion with the patient and their family, we decided to proceed with conversion surgery.

### Surgery

A right hemicolectomy with D3 lymphadenectomy was performed on Day 182 using an open approach. Dense fibrosis, likely induced by the tumor, was observed in the tissue between the anterior surface of the duodenum and the tumor. However, a dissectible layer was present between the fibrosis and the duodenum. This layer was carefully dissected using scissors without causing any injuries.

Functional end-to-end anastomosis was performed using linear staples. The surgery duration was 199 min (skin-to-skin), with 290 mL of blood loss. The postoperative course was uneventful, with no Grade 2 complications or higher, according to the Clavien-Dindo classification. Thereafter, the patient was discharged on Day 8 after surgery (Day 190). There has been no decline in ADLs since the surgery, and the patient lives independently in his own home.

There were no signs of recurrence at 10 months after operation. Regular follow-up observations will continue in the outpatient department.

### Final pathological diagnosis

**Fig. 6:**
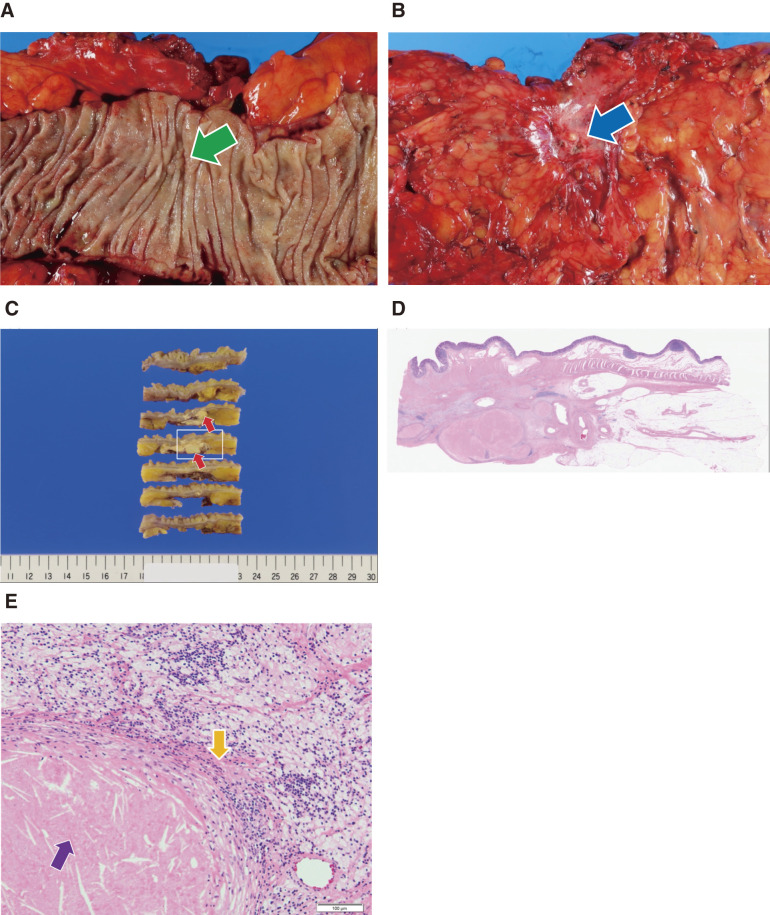
Macro and micro images of the specimen obtained after preoperative chemotherapy for ascending colon cancer. In the micro images, the arrows indicate representative areas, and the changes described may not be limited to a single location. (**A**) There was a 1 cm area of mucosal retraction in the ascending colon (green arrow). (**B**) Serosal surface: Scarring with serosa retraction is present (blue arrow). (**C**) Cross-sectional observation: A yellowish-white nodular change (red arrow) of approximately 2 cm, predominantly in the submucosal layer, was observed. (**D**) Low magnification image of the area marked by the white rectangle in (**C**). (**E**) Necrotic lesion suspected to be a consequence of preoperative chemotherapy (purple arrow). No residual viable adenocarcinoma components were observed. Fibrosis is noted in some areas (orange arrow).

Ascending colon, resection; colon cancer after chemotherapy; A, no residual carcinoma component; ypT0, Ly0, V0, PN0, pPM0, pDM0, pRM0, ypN0 (0/17), ypStage-0. Histological effect for chemotherapy; primary and lymph node, Grade 3. Macro and micro images of the specimen have been provided in **Fig. 6**.

## DISCUSSION

CRC is typically treated with en bloc resection of the colon and mesentery, with the aim of achieving a complete cure. En bloc resection of the organs involved is considered when the tumor directly invades the adjacent organs. However, when there is suspected infiltration into the duodenum and/or pancreas, simultaneous pancreaticoduodenectomy is necessary, raising concerns about the increased incidence of surgical complications. Adjuvant chemotherapy is required in cases of locally advanced disease; however, surgical complications may delay the timely initiation of chemotherapy, raising concerns about the potential for successful treatment. According to Kaneda et al., the 5-year survival rate following right hemicolectomy for pancreaticoduodenectomy is approximately 60%.^7)^ Yan et al. reported that the 5-year survival rate for patients with right-sided colon cancer with concurrent pancreaticoduodenectomy was comparable to that reported by Kaneda et al.^8)^ According to Yan et al., prognostic factors include the histological type, presence of lymph node metastasis, MSI status, and administration of postoperative chemotherapy.^8)^

Furthermore, one of the factors that leads to a hesitation in performing combined resection is the challenge in clearly distinguishing between inflammatory adhesions and the direct infiltration of malignant tumors using preoperative CT scans or intraoperative findings. In the aforementioned report by Kaneda et al., among the cases in which extended right hemicolectomy was conducted due to suspected infiltration, 20% did not show infiltration on histology.^7)^ Furthermore, Marubashi et al. reported that right-sided colon cancer has a high operative mortality rate (1.9%–2.5% of 90 days), and a high proportion of older adult patients (approximately 70%) are over 70 years old.^9)^

Therefore, based on pathological findings, it can be inferred that extensive resection of locally advanced CRC may result in unnecessary resection of adjacent organs, potentially increasing the frequency of surgical complications, and worsening the prognosis. In addition, reducing surgical invasiveness is desirable based on the chronological age range of the patients and the perioperative mortality rate. Neoadjuvant treatment, which promotes tumor shrinkage in locally advanced colon cancers suspected of directly invading adjacent organs, could be an effective strategy to reduce surgical invasiveness. In this study, although the postoperative findings suggested potential tumor infiltration to the adjacent duodenal wall before chemotherapy, tumor shrinkage was observed following preoperative immunotherapy, and R0 resection of the colon cancer was achieved, without pancreaticoduodenectomy. There were no major perioperative complications, and ADLs were maintained. Evaluation of the resected specimen confirmed a pathological complete response.

The proportion of MSI-high unresectable colon cancer cases in Japan is approximately 4%.^2)^ However, the frequency of MSI-high from Stage I to III has not been clearly established. Global reports indicate that the frequency approximately ranges from 10% to 20%, with such groups associated with better prognosis.^10)^ Furthermore, approximately 40% of MSI-high cases are reported to be positive for the *BRAF*^*V600E*^ mutation.^11)^

According to the KEYNOTE-177 study, complete response was achieved in approximately 13% of patients with Stage IV colon cancer.^12)^ In addition, the NICHE-1 study reported that pathological complete response was achieved in 60% of CRC cases with dMMR and no distant metastases upon treatment with neoadjuvant chemotherapy using ICIs.^4)^ Moreover, the NCT04165772 study documented a cCR rate of 100% following an observation period of ≤6 months for ICIs in the management of locally advanced rectal cancer with dMMR.^13)^ Therefore, ICIs may become an effective treatment option in the future, even for MSI-high CRC without distant metastasis.

A disadvantage of administering ICIs as neoadjuvant therapy for patients with Stage I to III cancer is the occurrence of irAEs, including interstitial pneumonitis or endocrine disorders. It is important to consider the possibility of missing the optimal timing of surgery in patients who could potentially be cured only with surgery. Delay in surgery due to irAEs occurred in approximately 3% of cases according to the NICHE-2 study, highlighting the need for further research in this area.^5)^

Furthermore, considering the possibility of achieving high rates of complete response with immunotherapy in patients with Stage I to III CRC, it is important to discuss the timing of MSI or dMMR measurements. Therefore, it may be necessary to reconsider treatment algorithms for CRC, including the measurement of these markers in preoperative biopsy samples. In this case, lower gastrointestinal endoscopy was promptly performed following the initial consultation, and genetic mutation analysis was conducted along with biopsy. However, the analysis results were not available before commencing the initial treatment. Consequently, mFOLFOX6 therapy was administered as an interim measure until the results were obtained, which ultimately was unimportant for this patient. This highlights the importance of genetic mutation analysis of preoperative biopsies to effectively tailor treatment strategies.

From the standpoint of organ preservation and minimally invasive procedures, a W&W approach could have been justified if significant tumor shrinkage and cCR had been confirmed through re-evaluation with endoscopy or supplementary MRI. Although the W&W approach is effective in rectal cancer after chemotherapy and radiotherapy, this strategy carries a 34% local recurrence rate, often requiring additional resection.^14)^ Although case reports exist, the efficacy and safety of the W&W approach in colon cancer after ICI with chemotherapy have not yet been established through rigorous scientific evidence.^15)^ In this case, the risk of losing the possibility of complete resection due to potential tumor regrowth, along with limited improvement in ADL, led us to decide to proceed with resection as initially planned.

This case report had some limitations. The report was based on a single-center case. However, ongoing clinical trials support the use of preoperative ICIs as an effective treatment strategy for CRC.

The uniqueness of our report is that immunotherapy for clinically unresectable locally advanced CRC allowed for the patient to undergo conversion surgery without adjacent organ resection, thereby minimizing the invasiveness of this surgery.

## CONCLUSIONS

We reported a case of locally advanced ascending colon cancer with MSI-high and *BRAF*^*V600E*^ mutation that achieved a pathological complete response to immunotherapy, without extended combined resection. Further evaluation of its efficacy and safety through the accumulation of additional cases is needed.

## ACKNOWLEDGMENTS

We would like to thank Editage (www.editage.jp) for English language editing.

## DECLARATIONS

### Funding

This study received no funding.

### Authors’ contributions

TN and YS designed this study.

TN wrote the initial draft of the paper.

YS supervised the study and revised the manuscript.

NH conducted the histopathological assessments.

TN, YS, NK, YM, KK, AS, KW, and MT were involved in the patient’s treatment.

All the authors have read and approved the final manuscript, and each author agrees to be held accountable for all aspects of the research.

### Availability of data and materials

Not applicable.

### Ethics approval and consent to participate

This work does not require ethical considerations or approval. Informed consent to participate in this study was obtained from the patient.

### Consent for publication

Informed consent for publication of this case report was obtained from the patient.

### Competing interests

The authors declare that they have no competing interests.
